# Anxiety in Myasthenia Gravis Patients Throughout the COVID‐19 Pandemic–Prevalence, Risk Factors, and Association With Vaccination Status

**DOI:** 10.1002/brb3.70878

**Published:** 2025-09-16

**Authors:** Janna Beckmann, Moritz Petzold, Felix Betzler, Andreas Ströhle, Antonia Bendau, Carla Dusemund, Gabor C. Petzold, Andreas Meisel, Sarah Hoffmann

**Affiliations:** ^1^ Department of Vascular Neurology University Hospital Bonn Bonn Germany; ^2^ Department of Psychiatry and Neurosciences Charité–Universitätsmedizin Berlin Corporate Member of Freie Universität Berlin and Humboldt Universität zu Berlin Berlin Germany; ^3^ Department of Psychology–Medical School Berlin Berlin Germany; ^4^ German Center for Neurodegenerative Diseases (DZNE) Helmholtz Association Bonn Germany; ^5^ Department of Neurology and NeuroCure Clinical Research Center Charité–Universitätsmedizin Berlin Corporate Member of Freie Universität Berlin and Humboldt‐Universität zu Berlin Berlin Germany

**Keywords:** autoimmune disease, corona, fear, immunosuppressive treatment

## Abstract

**Introduction:**

A high prevalence of COVID‐19‐related anxiety was observed among the general population during the COVID‐19 pandemic. Patients with myasthenia gravis (MG) might be at higher risk for COVID‐19‐related anxiety due to immunosuppressive therapy in the majority of cases and risk of myasthenic exacerbation in case of an infection. This prospective longitudinal study assessed COVID‐19‐related anxiety in MG patients over 2 years of the pandemic and its association with MG‐specific factors (e.g., disease severity, immunosuppressive medication) and with vaccination status.

**Methods:**

A three‐wave longitudinal online survey was conducted from May 2020 to February 2022, including a total of 648 adult MG patients. Descriptive, univariate, and multivariate analyses were performed to assess COVID‐19‐related and MG‐related anxiety, associated MG‐specific factors, and association with vaccination status.

**Results:**

COVID‐19‐related anxiety was frequent, with the most pronounced fear being risk for one's own health (overall 83.9%). Anxiety was influenced by MG diagnosis (overall 75.1%), especially by fear of exacerbation of myasthenic symptoms and greater risk of infection due to immunosuppressive therapy. Female sex and treatment with immunosuppressive medication, including rituximab, were associated with an increased concern for one's own health. Fear for one's own health was the only factor associated with vaccination status.

**Conclusions:**

COVID‐19‐related anxiety was prevalent among MG patients. An ongoing psychological impact is likely and highlights the need for anxiety screening in clinical practice. Our findings emphasize the importance of addressing mental health challenges during future pandemics.

## Graphical abstract

COVID‐19‐related anxiety is highly prevalent among patients with myasthenia gravis, and anxiety levels are influenced by the diagnosis of myasthenia gravis. Fear for one's own health is associated with positive vaccination status.

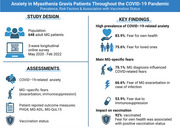

## 1 Introduction

The COVID‐19 pandemic has posed a global challenge with a significant impact on mental health (Qiu et al. 2020). Numerous studies have shown a high prevalence of COVID‐19‐related anxiety among the general population worldwide (Delpino et al. 2022; Santomauro et al. 2021). Contributing factors include uncertainty, disruptions in daily routines, and concerns for personal health, as well as the health of family members and loved ones (Delpino et al. 2022). In a German study conducted during the early months of the COVID‐19 pandemic, over 50% of the general population reported experiencing anxiety related to COVID‐19, with about 30% suffering from moderate‐to‐severe anxiety symptoms (Petzold et al. 2020b). As the pandemic progressed, the prevalence and severity of anxiety decreased, as shown in several studies (Bendau et al. 2022; Daly and Robinson 2021). Various risk factors for COVID‐19‐related anxiety have been proposed, with consistent findings for female sex, younger age, and preexisting mental or physical diseases (Bäuerle et al. 2020; Bendau et al. 2021; Gilbar et al. 2022; Guo et al. 2020; Hubbard et al. 2021; Santomauro et al. 2021). Myasthenia gravis (MG) is an antibody‐mediated autoimmune disease that affects the neuromuscular junction (NMJ) of skeletal muscles, leading to variable fatigable muscle weakness that can be life‐threatening if respiratory muscles are affected (myasthenic crisis in up to 20% of MG patients) (Nelke et al. 2022). A two‐way interaction made MG patients during the COVID‐19 pandemic a particularly vulnerable group: On the one hand, about 70% of MG patients require immunosuppressive treatment (IST), which leads to an immunocompromised state with increased risk of infection, as well as increased risk of a more severe disease course in the event of an infection (Alcantara et al. 2023; Kassardjian et al. 2020; Mevius et al. 2023). On the other hand, infections, and especially acute respiratory infections, are the most common triggers for potentially life‐threatening myasthenic exacerbations and myasthenic crisis (Gilhus et al. 2018; Gummi et al. 2019; Nelke et al. 2022; Neumann et al. 2020; Tugasworo et al. 2022). Therefore, MG patients may be at high risk for COVID‐19‐related anxiety, as shown for other neurological autoimmune diseases (Alnajashi and Jabbad 2020; Ramezani et al. 2021).

The aim of this longitudinal study was to assess the prevalence and degree of COVID‐19‐related anxiety in MG patients over the course of 2 years and its association with MG‐specific factors such as disease severity and IST. During the study period, COVID‐19 vaccines became available in Germany, and the study aim was expanded to assess the association of COVID‐19‐related anxiety with vaccination status.

## 2 Methods

### 2.1 Design, Recruitment, and Study Period

A longitudinal observational study with anonymized interviewing via online questionnaire during three survey periods (SP) was carried out between May 2020 and February 2022. Information on the study and the invitation to participate in the online survey via the link were distributed through four different channels: (1) On the website of the German Myasthenia Society (DMG, https://dmg‐online.de/), the largest German support group for patients with myasthenic syndromes with > 3000 members, (2) in the DMG patient journal “DMG‐Aktuell,” (3) at the largest certified Integrated Myasthenia Center (IMZ) in Germany, Charité ‐ University Medicine Berlin, treating > 2000 MG patients, and (4) outreach to moderators of German‐speaking MG support groups within the D‐A‐CH region (Germany, Austria, and Switzerland) on Facebook with the request to share the link within their community. Invitations for participation were sent out at three different times throughout the study period. SP1 lasted from May 28, 2020, to September 1, 2020; SP2 from November 29, 2020, to February 20, 2021; and SP3 from November 30, 2021, to February 7, 2022. Paid advertising was not used; no compensation was offered.

### 2.2 Ethical Statement

Informed consent was obtained from all participants included in the study (first page of online survey); survey completion took approximately 15 min. Data assessment was fully anonymous. Data were stored separately from contact information (i.e., email addresses) and merged via anonymous codes on the participant level. The study was approved by the ethics committee of the Charité‐ University Medicine Berlin, Germany (EA1/138/20).

### 2.3 Eligibility Criteria

Patients with the diagnosis of autoimmune MG, with a minimum age of 18 years and the ability to complete the questionnaire in German, were included in the study. Age and diagnosis had to be confirmed via checkbox before the actual questionnaire could start.

### 2.4 Assessment

An online survey via *SoSci Survey* was used to investigate prevalence and degree of anxiety, associated factors, and their association with vaccination status throughout the COVID‐19 pandemic. Completion of the entire survey required approximately 15 min. Sociodemographic data included age and sex (female, male, diverse). MG‐related questions included disease duration, antibody status (AchR, MuSK, LRP4, and seronegative), current MG‐specific medication (including cholinesterase inhibitors [ChEI], corticosteroids, long‐term immunosuppressants [azathioprine, mycophenolate mofetil, methotrexate, ciclosporine], escalation therapies (rituximab, eculizumab), acute therapies (intravenous immunoglobulins [IVIg], plasma exchange [PLEX], immunoadsorption [IA]), history of thymectomy and thymic histopathology, and patient‐reported outcome parameters (MG activities of daily living profile [MG‐ADL], MG‐specific quality of life instrument 15 [MG‐QoL15]) (Burns et al. 2008; Wolfe et al. 1999). Pandemic‐related information consisted of binary questions (yes/no) regarding being diagnosed with and/or hospitalized due to COVID‐19, as well as knowing someone who has been diagnosed with COVID‐19. COVID‐associated anxiety was assessed using a questionnaire already used in the German general population (Bendau et al. 2022; Petzold et al. 2020a) and included fear of being infected, fear concerning one's own health, fear concerning health of others (beloved ones), fears that are debilitating in everyday life, as well as fear of social (e.g., less contact with family and friends) or economic consequences (e.g., unemployment). All statements were rated on a 6‐point Likert scale, ranging from 1 (*not true at all*) to 6 (*totally true*). MG patients were then asked if COVID‐associated anxiety was influenced by MG (yes/no) and, in case of indicating “yes,” why (fear of increased risk of COVID‐19 infection due to MG itself, fear of increased risk of COVID‐19 infection due to MG medication [e.g., steroids, long‐term immunosuppression], fear of worsening of myasthenic symptoms in case of COVID‐19 infection, and fear regarding other aspects of MG). Patients could indicate multiple MG‐related fears. Symptoms of unspecific generalized anxiety (GAD‐2) and depression (PHQ‐2) were measured using the screening scale of the Patient Health Questionnaire‐4 (PHQ‐4). The four items are rated on a 4‐point scale, ranging from 0 (*not at all*) to 3 (*nearly every day*). The sum score is classified as normal (0–2), mild (3–5), moderate (6–8), or severe (9–12) (Löwe et al. 2010).

During the first two SPs, the same survey was used. In SP3, when COVID‐19 vaccines became available, questions on vaccination (yes/no), type of vaccine administered, and adverse events were added. We included information on vaccination status (yes/no) in the present analysis.

### 2.5 Statistical Analysis

All analyses were performed using R Statistical Software (Core Team 2022). The psych package was used for factor analysis (Revelle 2022). Alpha level was set to 0.05. Missing values within a sum score (MG‐ADL, MG‐QoL15) were imputed using the mean for single imputation. For the rest of data, missing values were excluded by a case‐by‐case deletion. The SP was divided into three timeframes, matching the three time periods of the invitation for participation mentioned above. The few questionnaires submitted in the meantime were assigned to the previous timeframe. Descriptive statistics were used for data analyses. Pearson's chi‐squared test and analyses of variance were performed to compare participant characteristics between the three SPs.

MG medication was classified into four groups, based on their effects: ChEI and ChEI extended release were grouped into symptomatic medication, and corticosteroids, azathioprine, mycophenolate mofetil, methotrexate, ciclosporine, and eculizumab were merged into immunosuppressive medication. Rituximab was analyzed separately to investigate its impact independently due to studies indicating a higher mortality in patients receiving rituximab, as well as a decreased likelihood to develop anti‐SARS‐CoV‐2 antibodies after COVID‐19 vaccination (Jakubíková et al. 2021; Yasuda et al. 2020). IVIg, PLEX, and IA were grouped as acute therapies.

To evaluate the prevalence of COVID‐associated anxiety, the answers to the items “fear of being infected,” “fear concerning one's own health,” “fear concerning health of beloved ones,” “fear of social consequences,” and “fear of economic consequences” were binary grouped into “disagree” and “agree.” The answers 1 (*not true at all*), 2 (*not true*), and 3 (*somewhat not true*) were counted as “disagree,” the answers 4 (*somewhat true*), 5 (*true*), and 6 (*totally true*) as “agree.”

After factor analysis, the items “fear of being infected with COVID‐19” and “fear of health consequences in case of infection” were combined into one factor, “fear for one's own health.” We a priori defined the following variables to be included in multivariate regression analysis for “fear for one's own health”: age, gender, disease severity as measured by the MG‐ADL and MG‐QoL15, as well as MG‐specific medication. We a priori defined the following variables to be included in multivariate regression analysis for vaccination status: age, gender, disease severity as measured by the MG‐ADL and MG‐QoL15, MG‐specific medication, as well as presence of “fear for one's own health.” We opted against including the MG‐QoL15 in multivariate regression analysis due to a high collinearity with the MG‐ADL (*r* = 0.77) according to current recommendations (Akinwande et al. 2015; Kim 2019). Multivariate linear regression was used to identify factors associated with “fear for one's own health” as a metric variable in SP1. Multivariable logistic regression was used to assess factors associated with vaccination (yes/no) as categorial variable in SP3.

## 3 Results

### 3.1 Participant Characteristics Throughout SP

**FIGURE 1:**
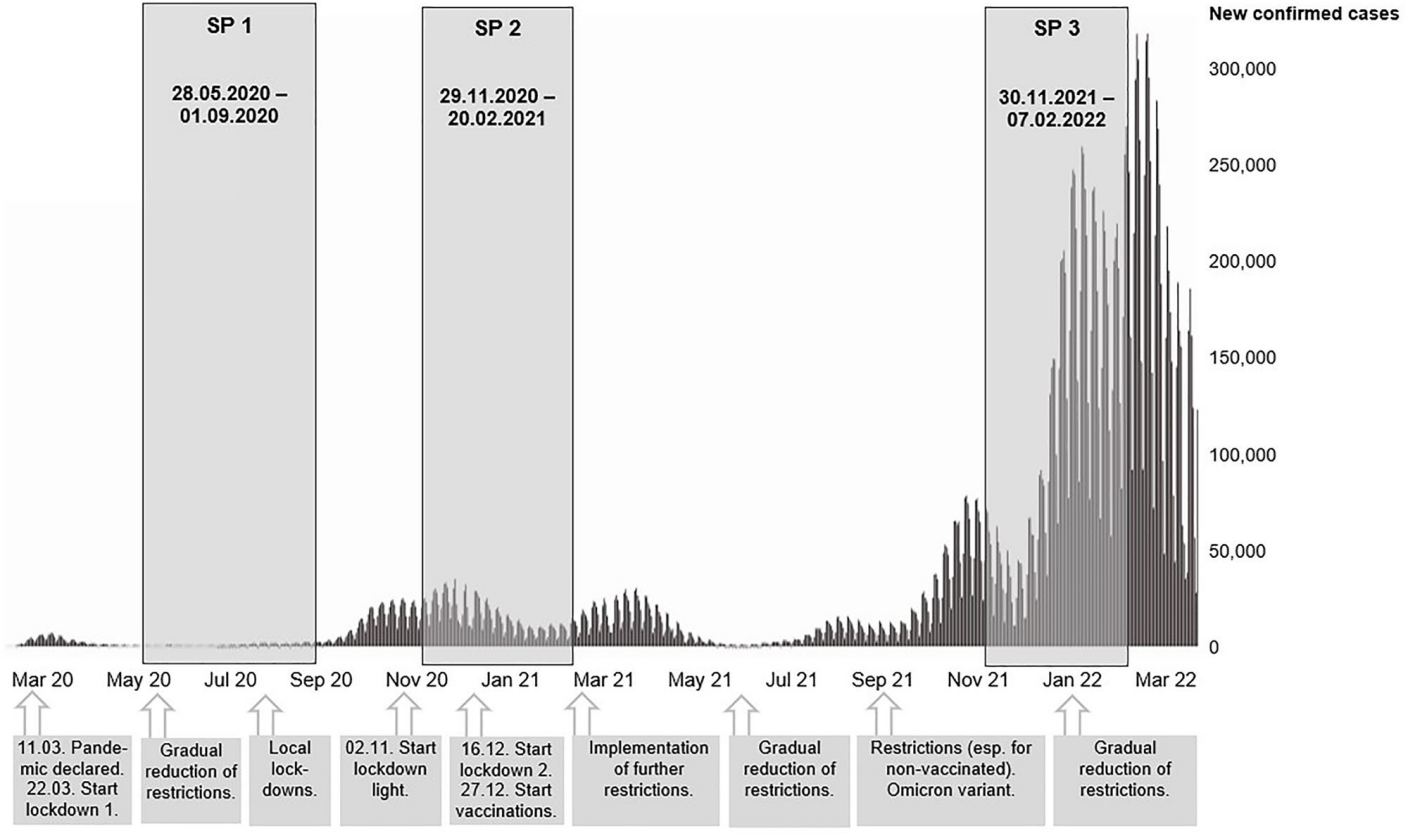
Survey periods (SP) in context to SARS‐CoV‐2 infection rates and pandemic‐related events in Germany.

Figure 1 provides an overview of the three SPs in context to the SARS‐CoV‐2 infection rates and pandemic‐related events in Germany. Participant characteristics for each SP are shown in Table 1. In SP1, 322 MG patients completed the survey, in SP2 295 and in SP3 392 MG patients, amounting to 1009 completed surveys overall. Out of 648 MG patients completing the survey overall, 401 MG patients participated once, 133 twice, and 114 MG patients took part in all three SPs. Over the course of all three SPs, mean age was 53.5 years (SD 15.1), 65.8% of patients identified as female, 34.2% as male, and none as diverse. 58.1% of patients were positive for acetylcholine receptor antibodies (AChR‐ab), followed by seronegative MG patients (22.7%), muscle‐specific tyrosine kinase (MuSK)‐ab (6%), and low‐density lipoprotein receptor‐related protein 4 (LRP4)‐ab (2.7%); in 17.4%, the ab‐status was unknown. Rate of thymectomy was 52.9%. The majority of patients were on immunosuppressive therapy (69.9%), and a smaller group received rituximab (7.8%). Mean disease severity as measured by MG‐ADL and MG‐QoL15 was 5 (SD = 3.9) and 19.4 (SD = 12.9), respectively. No significant differences were found in participant characteristics throughout the different SPs.

**TABLE 1:** Participant characteristics.

Characteristic	Survey period 1 May 2020 to September 2020	Survey period 2 November 2020 to February 2021	Survey period 3 November 2021 to February 2022	*p*‐value
*n*	322	295	392	
Sex, *n* (%)				
Female Male Divers Age, in years, mean (SD)	201 (62.4) 121 (37.6) 0 (0.0) 54.3 (15.2)	202 (68.5) 93 (31.5) 0 (0.0) 53.3 (15.2)	261 (66.6) 131 (33.4) 0 (0.0) 53.0 (15.1)	0.26 0.52
Disease duration, in years, mean (SD)	9.1 (9.3)	9.7 (9.8)	10.5 (10.7)	0.16
Antibody status, *n* (%)				
AchR	183 (56.8)	177 (60.0)	226 (57.7)	0.71
MuSK	25 (7.8)	15 (5.1)	21 (5.4)	0.29
LRP4	6 (1.9)	6 (2.0)	15 (3.8)	0.19
Seronegative	72 (22.4)	68 (23.1)	89 (22.7)	0.98
Unknown	59 (18.3)	50 (16.9)	67 (17.1)	0.88
Thymectomy, *n* (%)	165 (51.2)	164 (55.6)	205 (52.3)	0.53
Medication, *n* (%)				
Symptomatic medication	269 (83.5)	248 (84.1)	318 (81.1)	0.54
Immunosuppressive medication	234 (72.7)	205 (69.5)	266 (67.9)	0.37
Rituximab	24 (7.5)	22 (7.5)	33 (8.4)	0.86
Acute therapies	42 (13.0)	35 (11.9)	35 (8.9)	0.19
No medication	15 (4.7)	26 (8.8)	33 (8.4)	0.08
MG‐ADL, mean (SD)	5 (3.8)	5 (3.7)	5.1 (4.0)	0.91
MG‐QoL15, mean (SD)	19.4 (12.8)	19.0 (12.8)	19.6 (13.2)	0.84
PHQ‐4, mean (SD)	3.4 (3.4)	3.5 (3.2)	3.7 (3.1)	0.87
Vaccination, *n* (%)				
Yes No Not answered	n.a. n.a. n.a.	n.a. n.a. n.a.	362 (92.0) 24 (6.0) 6 (2.0)	

Abbreviations: AchR, acetylcholine receptor; LRP4, low‐density lipoprotein receptor‐related protein 4; MG‐ADL, MG activities of daily living; MG‐QoL15, MG‐specific quality of life instrument 15; MuSK, muscle‐specific tyrosine kinase; n.a., not applicable; PHQ‐4, patient health questionnaire‐4.

### 3.2 Exposure to COVID‐19

**FIGURE 2:**
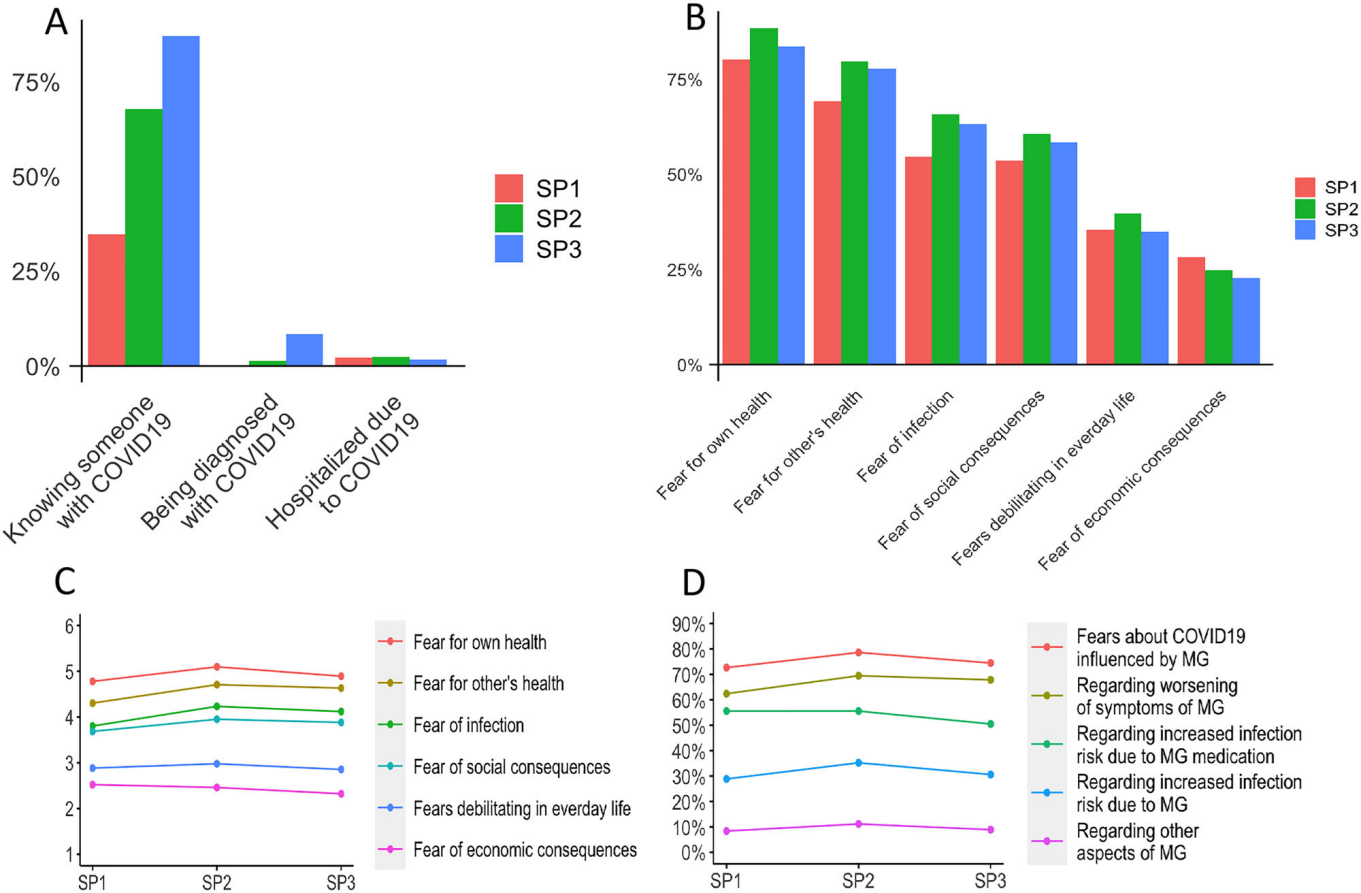
Exposure to COVID‐19 (A), prevalence (B) and degree (C) of pandemic‐related fears and prevalence of MG‐specific fears (D) throughout the COVID‐19 pandemic. Likert scale ranges from 1 (*no fear*) to 6 (*strong fear*); figure presents means (C). When indicating that COVID‐19 related fears were influenced by MG in general, multiple contents of MG related fears could be selected; figure presents frequencies (D). SP, survey period; SP1: May 2020 to September 2020 (n = 322); SP2: November 2020 to February 2021 (n = 295); SP3: November 2021 to February 2022 (n = 392).

The percentage of patients knowing someone who had been infected with COVID‐19 increased continuously from 34.8% in SP1 to 87.0% in SP3. The percentage of MG patients with COVID‐19 infection increased from 0.0% in SP1 to 8.4% in SP3. The percentage of MG patients being hospitalized due to COVID‐19 infection increased from 0.0% in SP1 to 1.8% in SP3 (Figure 2A).

### 3.3 Anxiety Regarding COVID‐19 and MG‐Related Anxiety

The degree of unspecific generalized anxiety and depressive symptoms as measured by the PHQ‐4 showed little variation throughout the study periods (overall mean = 3.5 [SD 3.2], Table 1).

The most prevalent *pandemic‐related* fear was the fear of negative consequences for one's own health in case of an infection (overall 83.9%), followed by fear for the health of others (overall 75.6%), fear of an infection in general (overall 61.2%), fear of social consequences (overall 61.2%), fears that are debilitating in everyday life (overall 36.5%), and fear of economic consequences (overall 25.1%, Figure 2B).

The most pronounced *COVID‐19‐related* fear was also the fear of negative consequences for one's own health in case of an infection (overall mean = 4.9 [SD 1.4]), followed again by fear for the health of others (overall mean = 4.5 [SD 1.5]), fear of an infection in general (overall mean = 4.0 [SD 1.6]), fear of social consequences (overall mean = 3.8 [SD 1.7]), fears that are debilitating in everyday life (overall mean = 2.9 [SD 1.6]), and fear of economic consequences (overall mean = 2.5 [SD 1.7], Figure 2C).

The majority of patients stated that their COVID‐19‐related fears were influenced by the diagnosis of MG (overall 75.1%). Most pronounced MG‐related fears concerned exacerbation of myasthenic symptoms in case of infection (overall 66.6%), followed by greater risk of infection due to myasthenic medication (overall 53.9%), greater risk of infection due to MG itself (overall 31.6%), and other aspects of MG (overall 9.5%, Figure 2D).

### 3.4 Factors Associated With COVID‐19 Pandemic‐Related Fears: Multivariate Analysis

Multivariate linear regression showed that female sex and treatment with immunosuppressive medication, as well as treatment with rituximab, were significantly associated with higher fear for one's own health (Table 2).

**TABLE 2:** Factors associated with “Fear for own health”: Multivariate linear regression.

Characteristic	Fear for own health Adjusted β‐coefficient (95% CI)	*p*‐value
Age	0.0 (−0.01 to 0.01)	0.81
Sex		
Male Female	Reference 0.51 (0.13 to 0.88)	< 0.01*
Disease duration	0.0 (−0.01 to 0.02)	0.38
MG‐ADL	−0.01 (−0.06 to 0.04)	0.69
Medication^a^		
Symptomatic medication	−0.29 (−0.78 to 0.2)	0.32
Immunosuppressive medication	0.7 (0.31 to 1.09)	< 0.01*
Rituximab	0.66 (0.05 to 1.27)	0.04*
Acute therapies	−0.09 (−0.59 to 0.41)	0.40
No medication	−0.87 (−1.77 to 0.03)	0.10

Abbreviation: MG‐ADL, myasthenia gravis activities of daily living.

^a^References in order: no symptomatic medication, no immunosuppressive medication, no rituximab, no acute therapies, any medication.

*Significant at 0.05‐level.

### 3.5 Vaccination Status and Associated Factors

The majority of MG patients (92.0%) received a vaccination when it became available after SP2 (Table 1). The only factor associated with being vaccinated was fear for one's own health. Sociodemographic and MG‐specific factors did not show an association with vaccination status (Table 3).

**TABLE 3:** Factors associated with vaccination status: Multivariate logistic regression.

Characteristic	Vaccination Adjusted OR (95% CI)	*p*‐value
Age	1.03 (0.99–1.06)	0.11
Sex		0.78
Male Female	Reference 0.85 (0.28–2.59)	
Duration	1.0 (0.95–1.04)	0.82
MG‐ADL	1.0 (0.88–1.13)	0.99
Fear for own health	1.71 (1.28–2.29)	< 0.01*
Medication		
Symptomatic medication Immunosuppressive medication Rituximab Acute therapies No medication	1.59 (0.42–6.05) 0.93 (0.3–2.93) 0.42 (0.1–1.74) 1.59 (0.18–14.37) 0.49 (0.08–3.01)	0.49 0.90 0.23 0.68 0.44

Abbreviation: MG‐ADL, myasthenia gravis activities of daily living.

*Significant at 0.05‐level.

## 4 Discussion

In this study, we show that COVID‐19‐related anxiety in MG patients was frequent, with the most pronounced fear being the risk for their own health. This anxiety is influenced by diagnosis of MG, especially by fear of exacerbation of myasthenic symptoms in case of infection and greater risk of infection due to immunosuppressive therapy. Furthermore, our findings indicate that female sex, treatment with immunosuppressive medication, and treatment with rituximab are associated with an increased fear for one's own health. Concerns for one's own health emerged as the sole statistically significant predictor of vaccination uptake.

The increased susceptibility to infection in case of immunosuppressive medication coupled with the potential of myasthenic exacerbation in case of an infection rendered MG patients particularly vulnerable during the COVID‐19 pandemic (Alcantara et al. 2023; Kassardjian et al. 2020; Mevius et al. 2023). This heightened vulnerability could also have extended to an increased risk of COVID‐19‐related fears, like in other neurological autoimmune diseases (Alnajashi and Jabbad 2020; Ramezani et al. 2021).

Our data show that a majority of MG patients experienced COVID‐19‐related fears, which targeted more personal (health‐related) rather than social aspects. Most and strongest fear concerned one's own health. These findings are consistent with previous studies, which indicated that anxiety regarding one's own health is common among individuals with chronic diseases, even before the pandemic (Lebel et al. 2020). In a study among the general population that used the same questionnaire, COVID‐19‐related fears were less prevalent, and they concerned mostly the health of others (Bendau et al. 2022).

In the majority of MG patients, anxiety was influenced by diagnosis of MG, particularly by fear of exacerbation of myasthenic symptoms in case of infection and greater risk of infection due to immunosuppressive therapy. Similar fears have been observed in patients with other autoimmune diseases, such as multiple sclerosis (MS) and rheumatoid arthritis (RA). In a smaller subset of MS and RA patients, the fear of an increased infection risk due to immunosuppressive therapy had significant clinical implications in both cohorts, particularly when patients changed their immunosuppressive medication themselves (Alnajashi and Jabbad 2020; Dahm et al. 2023).

Our findings also indicate that female sex, treatment with immunosuppressive medication, and the use of rituximab are associated with higher fear for one's own health. Anxiety is also more prevalent among women in the general population compared to men (Bäuerle et al. 2020; Bendau et al. 2021; Santomauro et al. 2021). Additionally, female MG patients tend to experience a higher disease severity (Dong et al. 2020; Lee et al. 2018; Lehnerer et al. 2022). These factors may reinforce each other.

In our cohort, unspecific generalized anxiety and depressive symptoms among MG patients were on average mild and comparable to the general population (Bendau et al. 2022). These results contrast with findings in other MG cohorts, as well as in patients with MS, where anxiety and depressive symptoms were common (Lehnerer et al. 2022; Zarghami et al. 2022). One possible explanation for this discrepancy is that the PHQ‐4, given its ultra‐brief format and limited item scope, may underestimate the actual prevalence or severity of psychiatric symptoms. However, prior studies have shown that the PHQ‐4 demonstrates acceptable psychometric properties, including in online applications, supporting its validity in digital survey contexts (Bisby et al. 2022; Löwe et al. 2010). Thus, while measurement limitations cannot be ruled out, they are unlikely to fully explain the lower symptom levels observed in our cohort. Another potential explanation is selection bias: participation in an online survey requires a certain degree of psychological stability, motivation, and energy. Patients with more pronounced psychiatric comorbidity may therefore have been less likely to participate, which could have resulted in an underrepresentation of individuals with higher psychiatric burden.

The percentage of MG patients hospitalized due to a COVID‐19 infection remained low despite a slight increase in infection rates over time. Infection rates in the general population were slightly higher compared to our MG cohort (Robert‐Koch‐Institut 2022a). This might be due to strict precautionary measures taken among MG patients (e.g., social distancing, wearing protective equipment, and avoiding travel) due to their higher risk profile, as reported in another study (De León‐Benedetti et al. 2021).

Vaccinations in Germany started on December 27, 2020 (Paul‐Ehrlich‐Institut n.d.). The limited number of vaccinations available in the beginning was triaged according to recommendations by the Standing Committee on Vaccination (STIKO) based on the risk for a severe or deadly disease course. Elderly were vaccinated in the early stages. Regardless of age, MG patients were at latest considered in stage 4 in the category “diseases of the central nervous system” (Robert‐Koch‐Institut 2021). The majority of patients in our cohort received their first vaccination in April 2021. Questions about vaccination were therefore included in the questionnaire in SP3, when it became widely available for our sample. Starting from June 2021, vaccinations were accessible for the general population (Bundesregierung 2021). By the end of our study in February 2022, the majority of MG patients (92%) were vaccinated. The primary factor associated with vaccination status was fear for one's own health, whereas other factors like disease severity and IST did not appear to have a significant association, potentially underscoring its pivotal role in patients’ health‐related decision‐making during the pandemic. These findings align with previous studies in the general population demonstrating that health‐related fears and fear of COVID‐19 were positively associated with willingness to get vaccinated and actual vaccination uptake (Bendau and Plag et al. 2021; Fadhel et al., 2024; Mertens et al. 2022). The vaccination rate in the general population was lower, at 76% (Robert‐Koch‐Institut 2022b), which might be partly explained by a lower degree of fear for one's own health in people in the general population. In addition to fear‐based mechanisms, other predictors identified in the literature include the vaccination behavior of close contacts, older age, and trust in governmental institutions, among others (Yan et al. 2022; Yuan et al. 2024).

Our study has several limitations that must be acknowledged. The correlational‐observational design does not allow for causal inferences. The sample was obtained through non‐probability convenience sampling, which may limit its representativeness for all MG patients, particularly older individuals, as the mean age was 53 years. Additionally, the reliance on online assessments may introduce selection bias. Participants who frequently use online media or have a particular interest in the subject matter are more likely to participate, while individuals with limited access to online platforms or lower digital literacy are likely underrepresented. Furthermore, making international comparisons is constrained by significant variability in pandemic conditions across different countries, including infection rates, public health restrictions, and available support measures (Daly and Robinson 2021; Prati and Mancini 2021). Moreover, there is considerable variation in the definition and assessment of psychopathological symptoms, such as anxiety, which can impact the results (Necho et al. 2021). It is also important to note that the data are based on anonymous self‐reports, which may be susceptible to response biases. As a result, the validity and reliability of the collected information may be limited, underscoring the need for supplementary objective data from clinical settings and practices.

In conclusion, our findings indicate that anxiety related to COVID‐19 was more prevalent among MG patients compared to the general population during the pandemic and mainly concerned one's own health. Recent data from a survey among the general population shows that although loneliness decreased over the course of the past years of the pandemic, health anxiety as well as depressive symptoms remained unchanged (Autenrieth et al. 2024). This makes an ongoing psychological and emotional impact of the COVID‐19 pandemic among MG patients likely and highlights the need for a screening for depression and anxiety in this population in clinical practice. Our data also underscores the importance of addressing mental health challenges in MG patients during future pandemics or other public health crises.

## Author Contributions

**Janna Beckmann**: Data curation, Investigation, Methodology, Formal analysis, Software, Validation, Visualization, Writing – original draft, Writing – review & editing. **Moritz Petzold**: Conceptualization, Investigation, Methodology, Resources, Writing – review & editing. **Felix Betzler**: Conceptualization, Investigation, Methodology, Resources, Writing – review & editing. **Andreas Ströhle**: Supervision, Validation, Methodology, Resources, Writing – review & editing. **Antonia Bendau**: Validation, Methodology, Resources, Visualization, Writing – review & editing. **Carla Dusemund**: Investigation, Validation, Writing – review & editing. **Gabor C. Petzold**: Supervision, Validation, Methodology, Resources, Writing – review & editing. **Andreas Meisel**: Supervision, Validation, Methodology, Resources, Writing – review & editing. **Sarah Hoffmann**: Conceptualization, Data curation, Investigation, Methodology, Project administration, Resources, Software, Supervision, Validation, Writing – original draft, Writing – review & editing

## Ethics Statement

The study was approved by the ethics committee of the Charité–Universitätsmedizin Berlin, Germany (EA1/138/20).

The authors assert that all procedures contributing to this work comply with the ethical standards of the relevant national and institutional committees on human experimentation and with the Helsinki Declaration of 1975, as revised in 2008.

## Consent

Informed consent was obtained from all participants included in the study.

## Conflicts of Interest

Felix Betzler has received speaker's honoraria and consulting fees from Takeda Pharmaceuticals and Medice. Andreas Meisel received speaker's speaker or consultancy honoraria or financial research support (paid to his institution) from Alexion AstraZeneca Rare Disease, argenx, Axunio, Desitin, Genpharm, Grifols, Hormosan, Immunovant, Janssen, Merck, Novartis, Octapharma, Regeneron, Sanofi, and UCB. He serves as member of the medical advisory board of the German Myasthenia Gravis Society. Sarah Hoffmann has received speaker's honoraria, consulting fees, or (institutional) financial research support from Alexion, argenx, UCB, Grifols, Novartis, Roche, and Johnson & Johnson and is member of the medical advisory board of the German Myasthenia Society. Remaining authors Janna Beckmann, Moritz Bruno Petzold, Antonia Bendau, Andreas Ströhle, and Gabor C. Petzold report no conflicts of interest.

## Peer Review

The peer review history for this article is available at https://publons.com/publon/10.1002/brb3.70878.

## Data Availability

Data will be shared at the request of any qualified investigator for purposes of replicating procedures and results.
